# Salinity-independent dissipation of antibiotics from flooded tropical soil: a microcosm study

**DOI:** 10.1038/s41598-020-70943-w

**Published:** 2020-08-24

**Authors:** Valerie Sentek, Gianna Braun, Melanie Braun, Zita Sebesvari, Fabrice G. Renaud, Michael Herbst, Katharina Frindte, Wulf Amelung

**Affiliations:** 1 Institute of Crop Science and Resource Conservation (INRES), Soil Science and Soil Ecology, University Bonn, Nussallee 13, 53115 Bonn, Germany; 2 Institute for Environment and Human Security (UNU-EHS), United Nations University, Platz der Vereinten Nationen 1, 53113 Bonn, Germany; 3 School of Interdisciplinary Studies, University of Glasgow, Dumfries Campus, Bankend Road, Dumfries, DG1 4ZL UK; 4 Institute for Bio- and Geosciences – IBG-3, Agrosphere, Forschungszentrum Jülich GmbH, 52425 Jülich, Germany; 5 Institute of Crop Science and Resource Conservation (INRES), Molecular Biology of the Rhizosphere, University Bonn, Nussallee 13, 53115 Bonn, Germany

**Keywords:** Biogeochemistry, Environmental sciences

## Abstract

River deltas are frequently facing salinity intrusion, thus challenging agricultural production in these areas. One adaption strategy to increasing salinity is shrimp production, which however, heavily relies on antibiotic usage. This study was performed to evaluate the effect of increasing salinity on the dissipation rates of antibiotics in tropical flooded soil systems. For this purpose, paddy top soil from a coastal Vietnamese delta was spiked with selected frequently used antibiotics (sulfadiazine, sulfamethazine, sulfamethoxazole, trimethoprim) and incubated with flood water of different salt concentrations (0, 10, 20 g L^−1^). Antibiotic concentrations were monitored in water and soil phases over a period of 112 days using liquid chromatography and tandem mass spectrometry. We found that sulfamethazine was the most persistent antibiotic in the flooded soil system (DT_50_ = 77 days), followed by sulfadiazine (DT_50_ = 53 days), trimethoprim (DT_50_ = 3 days) and sulfamethoxazole (DT_50_ = 1 days). With the exception of sulfamethoxazole, the apparent distribution coefficient increased significantly (*p* < 0.05) for all antibiotics in course of the incubation, which indicates an accumulation of antibiotics in soil. On a whole system basis, including soil and water into the assessment, there was no overall salinity effect on the dissipation rates of antibiotics, suggesting that common e-fate models remain valid under varying salinity.

## Introduction

Salinity intrusion is a natural phenomenon in coastal ecosystems that becomes particularly relevant in delta regions^1,2^. However, synergistic effects with anthropogenic drivers like groundwater overuse, hydro-dam construction, and sea level rise currently exacerbate salinization of deltaic soil and freshwater resources and restrict freshwater related agriculture^2,3^. In the Mekong Delta of Vietnam, for instance, salinization has already become a major threat^4^. About 1.8 million ha of land are affected by increasing salinity^5^, and the El Niño dry season 2015–2016 damaged up to 240,000 ha of paddy rice fields^6^. One approach to adapt to this changing environment is by shifting the freshwater land use systems, like paddy rice, to brackish or saltwater compatible agriculture, such as shrimp production^7^. Intensive shrimp production, however, frequently goes along with heavy use of antibiotics^8^, which disseminate in the environment. Accordingly, in shrimp ponds, single substances have already been detected in concentrations of up to 0.82 g kg^−1^ in pond mud and 2.39 mg L^−1^ in pond water^9^, thus posing risks for the selection and spread of antibiotic resistances^10^.

The final fate of antibiotics likely depends on soil order and temperature^11,12^, as well as several physico-chemical soil properties affecting sorption rates and dissipation^13,14^. Several studies dealt with dissipation rates of antibiotics in soil under field (e.g.^11,15^) or laboratory conditions (e.g.^16^^–^^18^). While field studies reflect natural conditions best, laboratory studies allow to determine the influence of a single factor on dissipation, in our case salinity, as environmental conditions can be fully controlled^11,19^. Results of such controlled experiments thus allow to estimate potential impacts of such factors on dissipation rates under field conditions^11,20.^ Respective studies, however have been performed mainly under freshwater conditions, such as studies on the fate of antibiotics in either soil^18,20^ or water phase^21,22^. Only very few studies investigated the fate of antibiotics in flooded soil systems or water–sediment systems^17,23,24^. Even fewer studies monitored antibiotics in saline environment^25,26^. Only for sulfonamides it has been reported that photodegradation in pond water was not affected by salinity while microbial degradation in river water and activated sludge was inhibited^22,27,28^. For trimethoprim it was reported that its adsorption was reduced under elevated salt concentrations of marine sediments and activated sludge of saline sewage^27,29^. However, we are not aware of studies that explicitly considered the impact of salinity on the fate of antibiotics in soil and flooded ecosystems as typically found in deltas.

Therefore, the aims of this study were: (i) to determine antibiotic dissipation rates in permanent flooded soil, and (ii) to investigate the effects of different salinity concentrations on the dissipation rates of antibiotics. For this purpose we conducted a microcosm experiment to quantify the effect of salt on antibiotic’s dissipation rates. The antibiotics sulfadiazine (SDZ), sulfamethazine (SMZ), sulfamethoxazole (SMX) and trimethoprim (TMP) were selected based on their use in Asian aquaculture (frequency of application and amount) derived by former studies^24,30^, and due to their relevance in human medicine as persistent antibiotics contributing to the selection and dissemination of resistance genes.

## Materials and methods

### Study site and soil

As soils from the coastal Mekong Delta were pre-contaminated with antibiotics^31,32^, we used soil from the coastal agricultural area of Nam Dinh province in the Red River Delta (Vietnam, 20° 15′ 39.28″ N, 106° 29′ 28.14″ E) for this study. When screening this soil for antibiotic residues prior to the experiment, none of our target antibiotics were detected. We thus deemed this soil as ideal for mechanistic studies on antibiotic dissipation in the lab. The texture of the soil was classified as silty clay loam (SiCL) according to^33^ with following composition: 3.7% (± 0.6) sand, 59.4% (± 1.9) silt and 38.5% (± 0.6) clay. The soil had a soil organic carbon (C_org_) content of 18.5 g kg^−1^ determined according to^34^, a pH_(H2O)_ of 7.95 determined after^35^, and cation exchange capacity of 10.8 cmol kg^−1^ (± 0.89) determined according to^36^. After the World reference base for soil resources^37^ the soil was classified as Fluvisol. After sampling, soil was air-dried and sieved to a grain size < 2 mm.

### Dissipation experiment

The dissipation experiment was conducted according to OECD Guideline 307^38^. Soil samples of 10 g were placed in centrifuges glasses and flooded with 25 mL water of three salinity concentrations (0 g L^−1^, 10 g L^−1^ and 20 g L^−1^). Salinity levels (10 g L^−1^ and 20 g L^−1^) were chosen to represent the salinity range in which shrimps (e.g. white leg or black tiger shrimp) can be cultured^7^. After a pre-incubation, water-soil systems were spiked with an antibiotic stock solution to achieve a target concentration of 60 µg L^−1^, respectively. The target concentration was chosen according to the routine limits of determination for the used antibiotics, adjusted to monitoring requirements for a dissipation study of 112 days. Antibiotic solutions for spiking were prepared in deionized water (prepared via Millipore purification system). After spiking, centrifuges glasses were covered with perforated aluminum foil to ensure air exchange and incubated in the dark at 25 °C for 0, 1, 3, 7, 14, 28, 56, and 112 days. Water-soil systems were prepared in triplicates for each extraction day and salt concentration. To verify microbial activity during experiment duration, respiration rates were determined for the incubation days 0, 56 and 112. Serum bottles were prepared in accordance with the incubation experiment in four repetitions per salt treatment and with and without antibiotic treatment. Respiration rates were determined by measuring the carbon dioxide concentration, using gas chromatography and flame ionization detector (GC-FID) (SRI, Torrance, USA) at the incubation day and the following day. The microbial activity was neither affected by the antibiotic treatment nor by the salt treatment (see Supplementary Table S1 online).

## Chemicals and reagents

All solvents used for extraction were of HPLC grade. Sand utilized for accelerated solvent extraction (ASE) and acids were proanalysis grade. The used water was purified with a Millipore water treatment system. Analytical standards of SDZ, SMZ, SMX and TMP as well as isotope-labeled standards of SMZ (SMZ-D_4_, purity ≥ 98%), SMX (ring-^13^C_6_, purity ≥ 98%), and TMP (methyl-^13^C_3_, purity ≥ 98%) were obtained from LGC Standards (Wesel, Germany).

## Sample extraction and purification

Antibiotics were analyzed separately in water, corresponding to easily extractable fraction, and soil, corresponding to the residual fraction^11,12,39^. Antibiotics were extracted in each system according to^12^.

Soil samples were extracted via accelerated solvent extraction (ASE; Dionex 350) using a methanol: water solution (1:1, v/v; according to^40^) and a 50 mM phosphoric acid: acetonitrile solution (50:50, v/v; according to^41^). The solutions used for ASE were adjusted to the water content of the samples.

Clean-up of the soil extracts and the water samples were performed using solid phase extraction (SPE). The samples were acidified with hydrochloric acid to pH 2.4 and loaded on the anion exchange cartridge (Chromabond SB, Macherey–Nagel, Düren, Germany) and adsorbent cartridge (OASIS HLB, Waters, Milford, United States). The antibiotics were eluted from the cartridges with 5 mL methanol, 5 mL acetonitrile and 5 mL of acidified acetonitrile (0.1% hydrochloric acid). Subsequently, the samples were evaporated to 0.5 mL in a rotary evaporator and filled up with 1 mL of 50 mM phosphoric acid: acetonitrile (80:20, v/v). Extracts were than stored at − 20 °C until analysis via liquid chromatography coupled to tandem mass spectrometry (LC/MS–MS). Laboratory blanks were taken regularly with each batch of samples.

### Antibiotic quantification

Antibiotic concentrations in extracts were analyzed using a Thermo Fisher system composed of a liquid chromatography coupled with TSQ Quantum Ultra tandem mass spectrometry (LC/MS–MS) (Thermo Fisher, Dreieich, Germany). The mass spectrometer was equipped with a heated electrospray ionization source (HESI), operating in positive mode. Antibiotics were separated by an XBridge C18 3.5 µm, 2.1 × 150 mm HPLC column with guard column Sentry 2.1 × 10 mm (Waters, Milford, MA, USA). The solvents used as mobile phases were methanol (A) and Millipore-water (B) both acidified with 0.1% formic acid. The routine limits of quantification (RLOQ) were 7 ng L^−1^ in water and 33 ng kg^−1^ in soil for SMZ, SMX and TMP. The RLOQ for SDZ were 33 ng L^−1^ in water and 165 ng kg^−1^ in soil (used mass to charge ratios are listed in Supplementary Table S2 online). The recovery of antibiotics from the water-soil systems were in the range of 48.8 to 100.3%.

### Data evaluation

For antibiotic dissipation calculations purposes, not detectable concentrations were set to zero and concentrations below RLOQ were set to the corresponding RLOQ. A single first-order exponential decay model (Eq. 1) and a first-order double-exponential decay model (Eq. 2) were fitted to the data, using nonlinear regression, where t is the time, C_t_ the antibiotic concentration at time t, C_0_ the concentration at time zero, C_1_ and C_2_ the constants for initial concentration of antibiotics in a fast and slow pool, respectively with k_1_ and k_2_ as the dissipation rate constants (k_1_ > k_2_).

1 $${C}_{t}={C}_{0}\cdot {e}^{-kt}$$

2 $${C}_{t}={C}_{1}\cdot {e}^{\left(-k1t\right)} + {C}_{2}\cdot {e}^{(-k2t)}$$

Prior to the fitting procedure the data was linearized by log-transformation in order to decide on the use of the single or double exponential-model^42^. The half-life DT_50_ (time required for 50% of the initial concentration to dissipate) was calculated using:

3 $$DT50=\mathrm{ln}\left(2\right)/k$$

Measured concentrations were normalized to their respective initial concentration to achieve consistency within the dissipation curves. Dissipation models were forced to the measured initial concentrations of the antibiotic accounting for differences in recovery.

To characterize the partitioning behavior of the antibiotics, apparent distribution coefficients (K_app_) were calculated for each extraction day as done by^11,39^. The K_app_ values were calculated at given sampling time using Eq. (4) (modified equation according to^11^).

4 $${K}_{app}= \frac{{C}_{soil}}{{C}_{water}}$$

For statistical analysis, IMP SPSS version 25.0 (IMB Corp, Armonk, New York, USA) and gnuplot version 5.2 were used. All applied statistical tests were run with *p* = 0.05, when not stated otherwise. The Shapiro–Wilk test was applied to test for normal distribution of the data. To find significant differences ANOVA or tow-sample t-test were performed and the Games-Howell test or R-E-G-W Q test were used for post-hoc tests. For not normally distributed data, the non-parametric tests Kruskal–Wallis H or Mann–Whitney U were used to identify differences between and within groups, respectively. Asymptotic standard errors were calculated to monitor differences between the three salinity concentrations within the model calculation. Overlapping asymptotic standard errors indicate that differences between the dissipation dynamics did not exist.

## Results

### Dissipation rates in flooded soil systems

**Figure 1:**
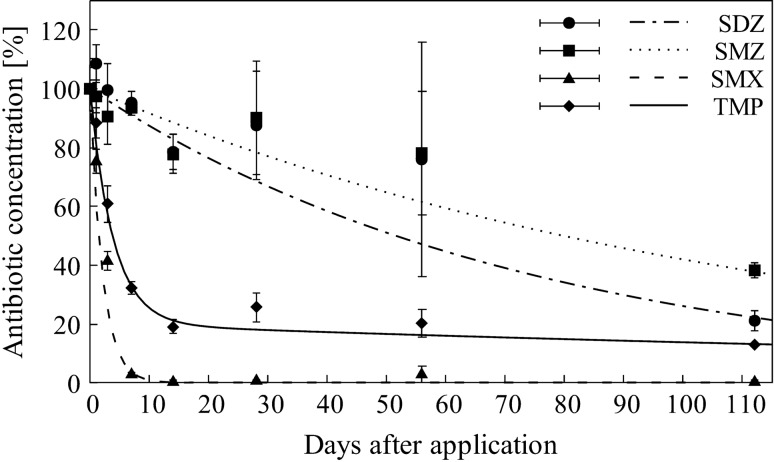
Dissipation of sulfadiazine (SDZ), sulfamethazine (SMZ), sulfamethoxazole (SMX) and trimethoprim (TMP) in a flooded soil system at 0 g L^−1^ salinity content (mean of three replicates ± standard deviation). Lines represent fitted dissipation curves.

**Figure 2:**
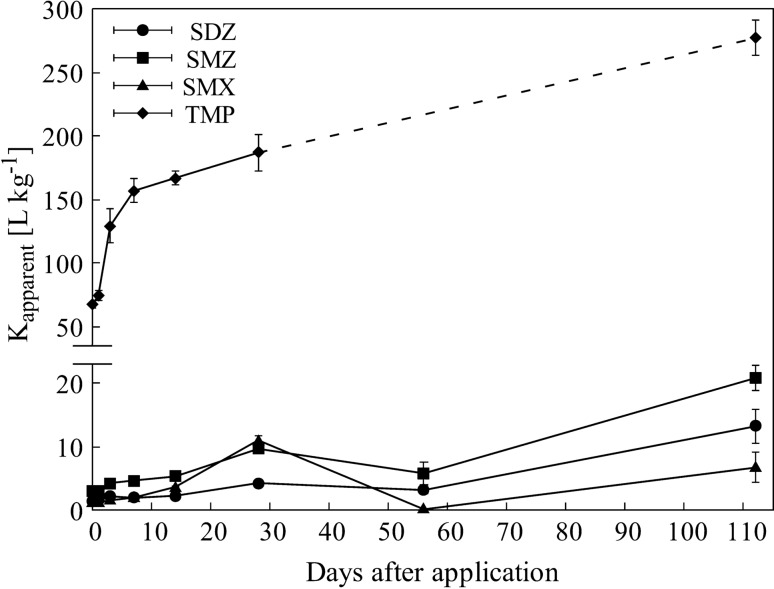
Apparent distribution coefficient (K_app_) of sulfadiazine (SDZ), sulfamethazine (SMZ), sulfamethoxazole (SMX) and trimethoprim (TMP) (mean of three replicates ± standard error) over the period of 112 days.

The dissipation of antibiotics in flooded soil systems followed single first-order kinetics for all studied sulfonamides and first-order double-exponential decay for TMP (Fig. 1). Dissimilarity in dissipation kinetics for TMP are due to the higher sorption affinity of TMP to the soil phase, identifiable by the calculated K_app_ values (Fig. 2). The coefficient of determination R^2^ (i.e. the “goodness-of-fit”) ranged between 0.84 (SDZ, SMZ) and 0.99 (TMP) (Table 1). Antibiotic concentrations decreased significantly for all antibiotics within 112 days (*p* < 0.01) in the order SMX (99.4%) > TMP (86.9%) > SDZ (78.9%) and > SMZ (61.9%) (Fig. 1). Corresponding dissipation half-lives (DT_50_) increased in the same direction and were 1 day for SMX and 3 days for TMP, while DT_50_ of SDZ (53 days) and SMZ (77 days), both being nearly 20 times higher than for SMX and TMP (Table 1).

**Table 1:** Fitting parameters of the dissipation models for sulfadiazine (SDZ), sulfamethazine (SMZ), sulfamethoxazole (SMX) and trimethoprim (TMP) for the water, soil and the total (water and soil) compartments for all salt concentrations; R^2^ (coefficient of determination); k (dissipation rate constant); DT_50_ (dissipation time for 50% of antibiotic concentration).

Antibiotic	Compartment	Salt (g L^−1^)	k	DT_50_ (days)	R^2^	Asymptotic standard error
SDZ	Water	0	0.035	19.80	0.89	0.004
		10	0.042	16.50	0.89	0.002
		20	0.045	15.40	0.98	0.003
	Soil	0	0.009	77.02	0.75	0.002
		10	0.010	69.31	0.75	0.003
		20	0.008	86.64	0.78	0.001
	Total	0	0.013	53.32	0.84	0.001
		10	0.012	57.76	0.84	0.025
		20	0.014	49.51	0.94	0.003
SMZ	Water	0	0.048	14.44	0.75	0.008
		10	0.056	12.38	0.82	0.006
		20	0.047	14.75	0.97	0.003
	Soil	0	0.006	115.52	0.73	< 0.001
		10	0.007	99.02	0.88	< 0.001
		20	0.007	99.02	0.85	0.002
	Total	0	0.009	77.02	0.84	< 0.001
		10	0.010	69.31	0.89	< 0.001
		20	0.009	77.02	0.90	0.003
SMX	Water	0	0.508	1.36	0.98	0.038
		10	0.352	1.97	1.00	0.012
		20	0.476	1.46	1.00	0.028
	Soil	0	0.412	1.68	0.98	0.068
		10	0.271	2.56	0.98	0.03
		20	0.388	1.79	1.00	0.016
	Total	0	0.470	1.47	0.97	0.067
		10	0.296	2.34	0.99	0.027
		20	0.435	1.59	1.00	0.020
TMP	Water	0	0.380	1.82	0.99	0.042
		10	0.165	4.20	0.93	0.113
		20	0.333	2.08	0.98	0.071
	Soil	0	0.273	2.54	0.98	0.035
		10	0.192	3.6	0.88	0.070
		20	0.279	2.48	0.88	0.093
	Total	0	0.256	2.71	0.99	0.020
		10	0.215	3.22	0.87	0.091
		20	0.304	2.28	0.88	0.120

*SDZ* sulfadiazine, *SMZ* sulfamethazine, *SMX* sulfamethoxazole, *TMP* trimethoprim.

The partitioning of antibiotics between the water and soil phase changed over the incubation period. The relative amounts of antibiotics occurring in soil increased with increasing contact time, which is illustrated by an increase in K_app_ values. Immediately after spiking (day 0), K_app_ values were comparatively highest for TMP (67.4 ± 2.87 L kg^−1^) followed by SMZ (3.1 ± 0.13 L kg^−1^), SDZ (1.4 ± 0.05 L kg^−1^) and SMX (1.2 ± 0.06 L kg^−1^) (Fig. 2). After 112 days, K_app_ values had increased significantly for SDZ (13.2 ± 2.66 L kg^−1^; p < 0.05), SMZ (20.8 ± 1.90 L kg^−1^; p < 0.05), and TMP (276.9 ± 13.92 L kg^−1^; p < 0.01) (Fig. 2).

### Effect of salinity concentrations on dissipation rates

According to their dissipation profiles, the studied sulfonamides can be divided into two groups: SDZ and SMZ exhibited similar DT_50_ values in both soil and water phase, differing to DT_50_ values obtained for SMX (Table 1). Thus, in the following we concentrate on illustrating only representative dissipation processes of SDZ and SMX for sulfonamide dissipation, and show dissipation data for SMZ in the Supplementary (Table S2 online). Due to significant increases of the K_app_ values, we discuss water and soil phase separately.

**Figure 3:**
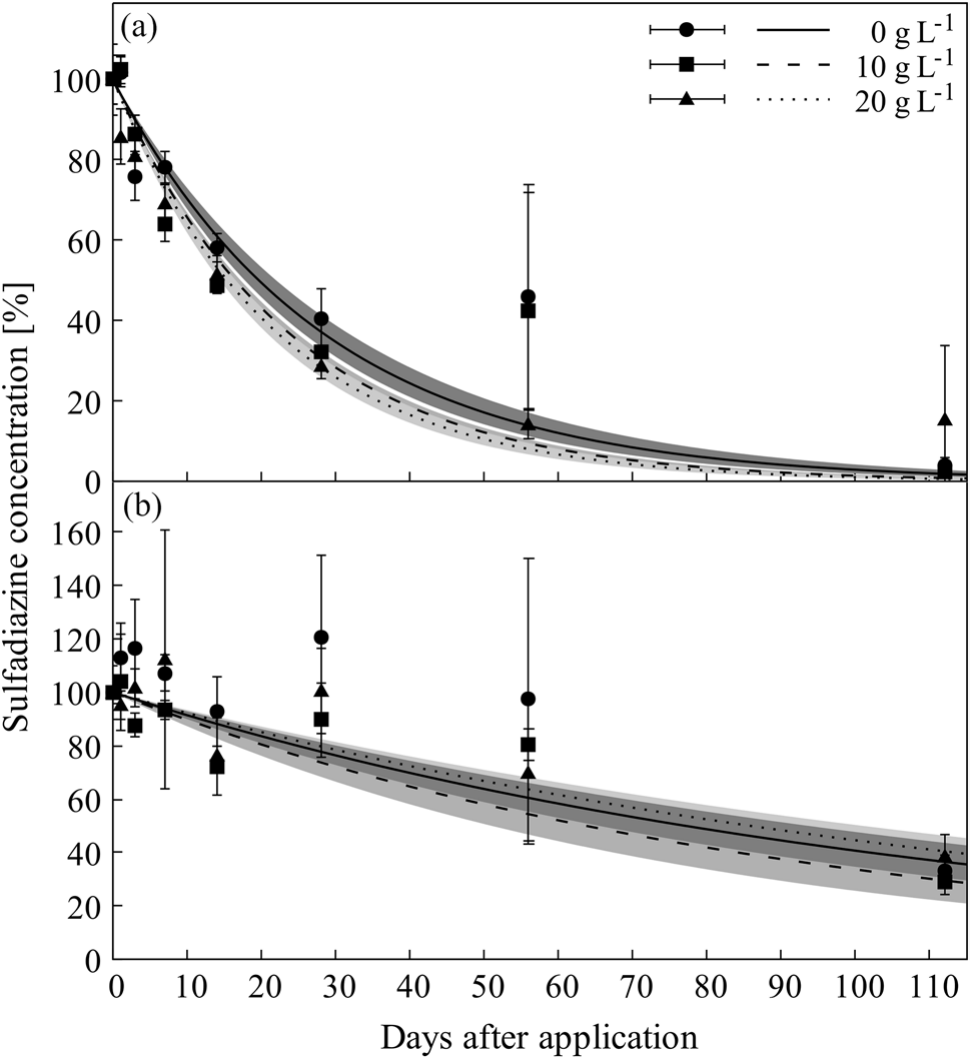
Dissipation of sulfadiazine in water (**a**) and soil (**b**) phase of flooded soil systems under different salt concentrations (0 g L^−1^, 10 g L^−1^, 20 g L^−1^). Data points and error bars represent the mean of three replicates with standard deviation; lines represent fitted dissipation curves; shaded areas represent respective asymptotic standard errors.

The R^2^ for the fitting of the dissipation curves ranged between 0.75 (SMZ) and 1.00 (SMX) for the water phase, and from 0.73 (SMZ) to 1.00 (SMX) for the soil phase (Table 1). Sulfadiazine dissipated faster in water than in soil, regardless of the prevailing salt concentration. The DT_50_ values ranged from a minimum of 15 days in water to a maximum of 87 days in soil (Table 1). The dissipation rates in water differed between the salinity levels, with a slower dissipation in the system without salt and DT_50_ values following the order 0 g L^−1^ (DT_50_ = 20 days) > 10 g L^−1^ (DT_50_ = 17 days) > 20 g L^−1^ (DT_50_ = 15 days; Table 1, Fig. 3a). SDZ dissipation in soil did not differ between the three salinity concentrations and ranged from 69 to 87 days, respectively (Fig. 3b, Table 1). The range observed in soil was larger than the difference observed for the water phase, reflecting than on a whole treatment basis (soil + water) no statistical differences were found among salinity levels (Table 1).

**Figure 4:**
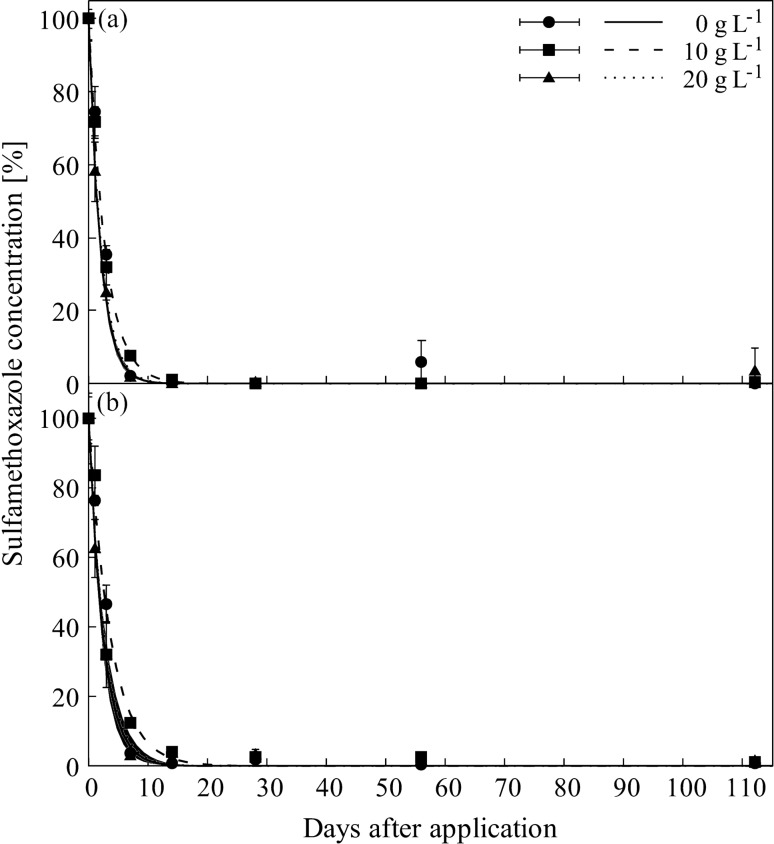
Dissipation of sulfamethoxazole in water (**a**) and soil (**b**) phase of flooded soil systems under different salt concentrations (0 g L^−1^, 10 g L^−1^, 20 g L^−1^). Data points and error bars represent the mean of three replicates with standard deviation; lines represent fitted dissipation curves; shaded areas represent respective asymptotic standard errors.

In contrast to SDZ (and SMZ; see Supplementary Fig. S1 online), the concentration of SMX decreased rapidly within the first 14 days for all salinity concentrations in the water and soil phase (Fig. 4, Table 1). Differences in dissipation rates between the three salinity levels were identified for SMX in water and also in soil phase. But in contrast to SDZ, dissipation of SMX was slowest at the intermediate salt concentration of 10 g L^−1^ compared to the other salinity levels (Fig. 4, Table 1). These differences in dissipation dynamics were mainly due to non-overlapping asymptotic standard errors between the incubation days 3 and 7 (Fig. 4), and possibly caused by chance (i.e., type I error), because absolute differences in DT_50_ values were smaller than a day. Furthermore, there is no real logical explanation for delayed dissipation at intermediate salinity levels, only.

**Figure 5:**
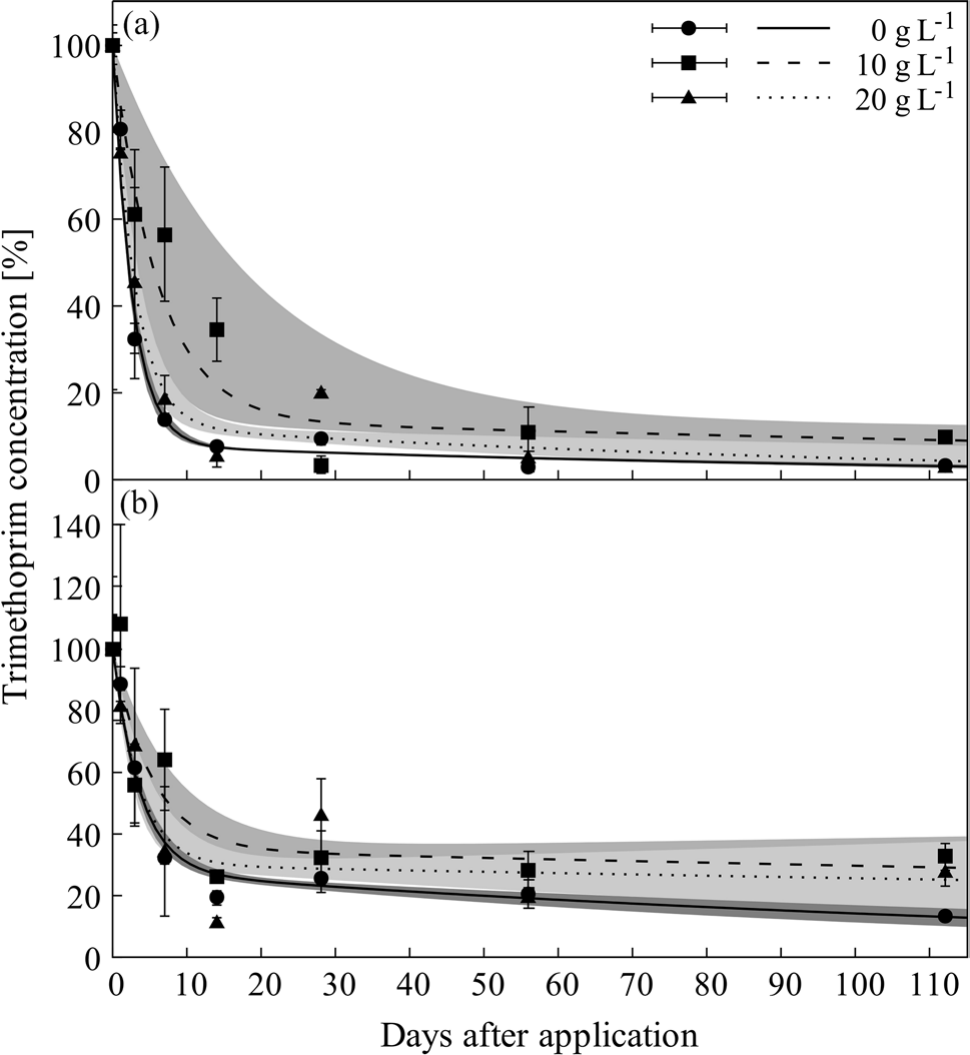
Dissipation of trimethoprim in water (**a**) and soil (**b**) phase of flooded soil systems under different salt concentrations (0 g L^−1^, 10 g L^−1^, 20 g L^−1^). Data points and error bars represent the mean of three replicates with standard deviation; lines represent fitted dissipation curves; shaded areas represent respective asymptotic standard errors.

The dissipation of TMP in water and soil phase deviated from that of the sulfonamides. Within the first days after application a rapid decline in TMP concentration was observed for both compartments, followed by a decelerated concentration decline after 7 to 14 days (Fig. 5). As a result, a first-order double-exponential decay model was needed to describe the dissipation behavior of TMP. In water, dissipation rates differed between the three salinity levels: dissipation at 0 g L^−1^ salt (DT_50_ = 2 days) was nearly two times faster than in the systems with 10 g L^−1^ salt (DT_50_ = 4 days; Fig. 5a; Table 1). This effect could not be confirmed for soil phase, where TMP dissipation did not differ between the three salinity levels, with DT_50_ ranging between 2 and 4 days (Table 1, Fig. 5b). Summing both phases together did not sustain statistical differences of the dissipation rates between the three salinity levels (Table 1).

## Discussion

The obtained DT_50_ value of SDZ in the flooded soil system (53 days Table 1) was higher than that reported of^24^ in a water–sediment system of a mesocosm study (DT_50_ = 32 days). The temperature during the mesocosm study did not exceed 30 °C in the water, while in our experiment the ambient temperature was 25 °C. Studies of^43^ as well as^44^ reported that sulfonamide dissipation and temperature were correlated, with slower dissipation rates at decreasing temperature. Beside this^11^, observed systematically faster antibiotic dissipation under field conditions compared to laboratory conditions, which could at least in parts be forecasted using temperature-dependent DT_50_ values.

For SMZ the DT_50_ in the flooded soil system was 77 days, which was also higher than derived from DT_50_ values estimated in soil solely (DT_50_ = 19–35 days^20,45^). A higher persistence of SMZ might be due to sequestration processes that shift extractable antibiotic residues into a residual soil fraction while reducing the bioavailable one prone to microbial degradation^10^. This suggestion finds support by K_app_ values that reached largest values for SMZ of all target sulfonamides (Fig. 2). A large potential for sulfonamides to be sequestered in soil has already been reported for the structurally related compound SDZ^11,46^.

In contrast to SDZ and SMZ, the rapid dissipation of SMX (DT_50_ = 1 days) was in agreement with previous findings under non-flooded conditions:^16^ found a rapid dissipation of SMX in soil within the first three incubation days (DT_50_ = 2–7 days) and^47^ observed up to 80% SMX dissipation within the 20 days of the experiment in loamy soil.

Despite the physico-chemical properties (listed in Supplementary Table S3 online) of sulfonamides indicate a rather weak sorption (log K_ow_ SDZ = − 0.09 and SMZ = 0.28) and high mobility and bioavailability in the soil^16^, it has been noted that both SDZ and SMZ may form non-extractable residues (NER), especially in samples with high soil organic carbon contents^17,48^. The elevated organic carbon concentration (18.5 g kg^−1^) of the experiment soil gives support to the hypotheses that similar processes likely took place here. However, to detect NER formation, it would have been required to work with radiolabeled organic compounds. For the used sulfonamides, microbial degradation plays the major role in the dissipation process^16,28^ while chemical processes like hydrolysis were negligible^16,22,48^.

Previously, a DT_50_ value for TMP of 8 days in a water–sediment system had been reported^24^, thus exceeding estimated DT_50_ values for TMP in our experiment (DT_50_ = 3 days; Table 1). Here, we have to recall that TMP dissipation rates were calculated using a first-order double-exponential decay model, which includes the risk that it underestimates the chemical´s persistence substantially^49^. Other researchers observed a bi-phasic dissipation only for hydrophobic pesticides^50^, assuming a slower second-phase dissipation process due to the higher affinity to soil sorption. Among the compounds studied here, TMP had the highest K_d_ values of all target antibiotic in this study and additionally, the pronounced increase in K_app_ values supports the idea that larger fractions of TMP were adsorbed to soil than for the sulfonamides (Fig. 2, Supplementary Table S4 online). The dissipation of TMP is usually controlled by biotic factors^16,47^. Abiotic factors like photolysis or hydrolysis can again be excluded, as the experiment were conducted in the dark and TMP does not consists of any hydrolysable groups or substituted amino groups^16^.

With the exception of SMX, all antibiotics accumulated in the soil during the experiment as indicated by the significant increase of the K_app_ values (SDZ, SMZ: *p* < 0.05; TMP: *p* < 0.01; Fig. 2). This is in line with^39^ and^11^ who observed increasing K_app_ values for SDZ within an incubation period of 218 and 288 days, respectively. Increasing K_app_ values were also reported in the context of an increase in the residual fraction, which was characteristic for sequestration processes^11,46^. Differences to SDZ and SMZ might be attributed to deviations in chemical structure. Although SMX, SZD and SMZ belong to the same antibiotic class sulfonamides show differences in the environmental fate. For instance^51^, reported that the sorption of sulfonamides can be largely affected by differences in functional moieties. Since SDZ and SMZ have more similarities in their chemical structure than with SMX, this might explain the deviations in the partitioning behavior between the used sulfonamides. Also TMP showed an increasing affinity to the soil, with K_app_ values more than 50 times higher compared to the sulfonamides, even at the first day of incubation (Fig. 2).

The above-mentioned discussion refers to the combined dissipation of our target compounds from soil and water phase together. When separating these two for SDZ, the dissipation was faster in the water phase (DT_50_ = 15–20 days; Table 1) than in other systems that solely investigated the dissipation of SDZ in water (DT_50_ < 120 days^28^; see also^23,24^). The main reason is likely the provision of additional soil surfaces for bacterial growth^16,23,28^. In soil, SDZ was found to be more persistent (DT_50_ = 69–86 days) than in the water phase (Fig. 3b), likely because of the formation of sequestered SDZ residues^39,46^, which have been included into our fractionation scheme.

When solely considering the water phase, SZD dissipation rates increased under the impact of higher salt concentration with a difference of 4 days in DT_50_ values between the systems with 0 g L^−1^ and those with 20 g L^−1^ salt (Fig. 3a). The main dissipation processes for SDZ in this experiment is assumed to be microbial degradation and sequestration. The former, however, should be reduced rather than enhanced in the salt-affected systems^27^, due to higher osmolality outside the cell^52–54^. As the soil used for this study originated from a coastal area, we may not fully discount the possibility that some members of the microbial community had been pre-exposed to salinity and thus were capable to adapt to a certain extent to this salinity^55^. Although the currently use of the soil for freshwater paddy rice production is not really in favor for this assumption. However, salinity has been reported to decrease sorption of antibiotics, particularly of those that bind to the solid soil matrix via ionic interaction to negatively charged surfaces^27,29^. Tropical soils frequently exhibit anion exchange capacity^56^ and there is no reason to ignore the possibility that in a similar manner also the anionic speciation of SDZ at the ambient pH may compete with accompanying chlorides and sulfates for sorption sites. In either case, the majority of SDZ primary binds to solid matrix via hydrophobic interactions^51,57^. This fraction is likely not affected by salinity, and as a result the overall effect of salts on SDZ dissipation in the water phase was low and absent for the soil phase.

The salinity effect observed for SDZ dissipation was not detected for SMZ (see Supplementary Fig. S1 online). Additionally, differences in the dissipation dynamics between the three salt treatments were not continuous over the whole incubation period. Moreover, for the rapidly dissipating antibiotic SMX, salinity effects on reduced dissipation rates were observed in the systems treated with 10 g L^−1^ salt, only (Table 1). As already indicated in the results section, there is no reasonable explanation that only intermediate salt concentrations might affect dissipation rates, so that this finding might well be a so-called alpha error in the statistical comparison. When taking into consideration that effects on DT_50_ were smaller than a day, the finding is also not relevant. For TMP, differences between the salinity levels were also limited to water phase and not continuous (Fig. 5). But this differences were negligible, as dissipation was not affected on a whole system basis, due to TMP mainly occurred in soil phase of the system (see K_app_ values, Fig. 2). The sorption of TMP to marine sediments was investigated by^29^ and they observed lower TMP sorption under the influence of salt. Indeed, we also found lower K_app_ values for TMP in the salt treated variants (10 and 20 g L^−1^) compared to the non-salt treated variant (0 g L^−1^) (see Supplementary Table S4 online). However, this finding did not affect TMP dissipation rates in soil. Overall, we thus have to refute the hypothesis that salinity exerts significant impacts on the dissipation rates of antibiotics in flooded soils.

## Conclusion

Within this study we investigated the dissipation of antibiotics in a permanently flooded tropical soil under different levels of salinity. Sulfonamide dissipation rates could be described via first-order exponential decay model, while TMP dissipation rates were calculated via first-order double-exponential decay model due to higher sorption affinity. SMZ and SDZ were the most persistent antibiotics, the fate of which being influenced by sequestration processes. Particularly for SDZ, SMZ, and TMP, there was an enhanced partitioning between soil and water phase over the incubation period with an increasing accrual of the antibiotics in flooded soil. However, an overall effect of salinity on the dissipation rates of antibiotics could not be ascertained. Hence, existing e-fate models from terrestrial environment remain valid and thus may continued to be used to forecast the fate of at least selected antibiotics also under conditions of salinity intrusion in such river deltas.

## Supplementary information

Supplementary Information.
