# Supplementary material for: Salinity-independent dissipation of antibiotics from flooded tropical soil: a microcosm study
Source: Sci Rep. 2020 Aug 24;10:14088. doi: 10.1038/s41598-020-70943-w (PMC7445273; doi:10.1038/s41598-020-70943-w)
Supplement: Supplementary file 1 — Supplementary Information. [file 41598_2020_70943_MOESM1_ESM.pdf]

Supplementary Material

Salinity-independent dissipation of antibiotics from flooded tropical soil – a microcosm study

Valerie Sentek[1*]; Gianna Braun[2*]; Melanie Braun[1]; Zita Sebesvari[2]; Fabrice G. Renaud [3]; Michael Herbst[4]; Katharina Frindte[5]; Wulf Amelung[1]

[1] Institute of Crop Science and Resource Conservation (INRES), Soil Science and Soil Ecology, University Bonn, Nussallee 13, 53115 Bonn, Germany

[2] Institute for Environment and Human Security (UNU-EHS), United Nations University, Platz der Vereinten Nationen 1, 53113, Bonn, Germany

[3] School of Interdisciplinary Studies, University of Glasgow, Dumfries Campus, Bankend Road DG1 4ZL, United Kingdom

[4] Institute for Bio- and Geosciences – IBG-3, Agrosphere, Forschungszentrum Jülich GmbH, 52425 Jülich, Germany

[5] Institute of Crop Science and Resource Conservation (INRES), Molecular Biology of the Rhiosphere, University Bonn, Nussallee 13, 53115 Bonn, Germany

*both authors contributed equally to this work

Corresponding author:

Valerie Sentek, phone: 0049 228 73 9368, fax: 0049 228 73 2782, mail: valerie.sentek@uni-bonn.de

Supplementary Tab. S1 Microbial respiration rates (mean of four replicates ± standard error) measured at three times over the period of 112 days under different salinity levels and antibiotic treatments; respiration rates were related to the soil wet weight

	Respiration rates [CO_2 nmol h^{-1} g^{-1} soil]					
	treated without antibiotics			treated with antibiotics		
**Salt**	0 days	56 days	112 days	0 days	56 days	112 days
0 g L^{-1}	26.42 (± 0.73)	12.06 (± 0.92)	4.95 (± 0.33)	25.98 (± 1.8)	8.42 (± 0.61)	5.8 (± 0.59)
10 g L^{-1}	39.57 (± 3.73)	11.51 (± 0.36)	3.60 (± 0.26)	38.28 (± 1.59)	12.04 (± 0.08)	3.28 (± 1.03)
20 g L^{-1}	40.21 (± 1.64)	10.05 (± 0.62)	3.03 (± 0.20)	41.47 (± 0.28)	10.98 (± 0.38)	3.37 (± 0.53)

Supplementary Tab. S2 Mass to charge ratios of antibiotics used for analysis via mass spectrometer; highlighted product ions were used for qualification and quantification

**Substance**	**Parent ion [m z^{-1}]**	**Product ion [m z^{-1}]**
SDZ	251	92, **108**, 156
SMZ	279	108, **124**, 186
SMZ-D_4	283	112, 124, **160**
SMX	254	108, **156**
SMX-$^{13}C_6$	260	98. **162**, 166
TMP	291	275, **230**, 261
TMP-$^{13}C_3$	294	**231**, 262, 277

SDZ, sulfadiazine; SMZ, sulfamethazine; SMX, sulfamethoxazole; TMP, trimethoprim

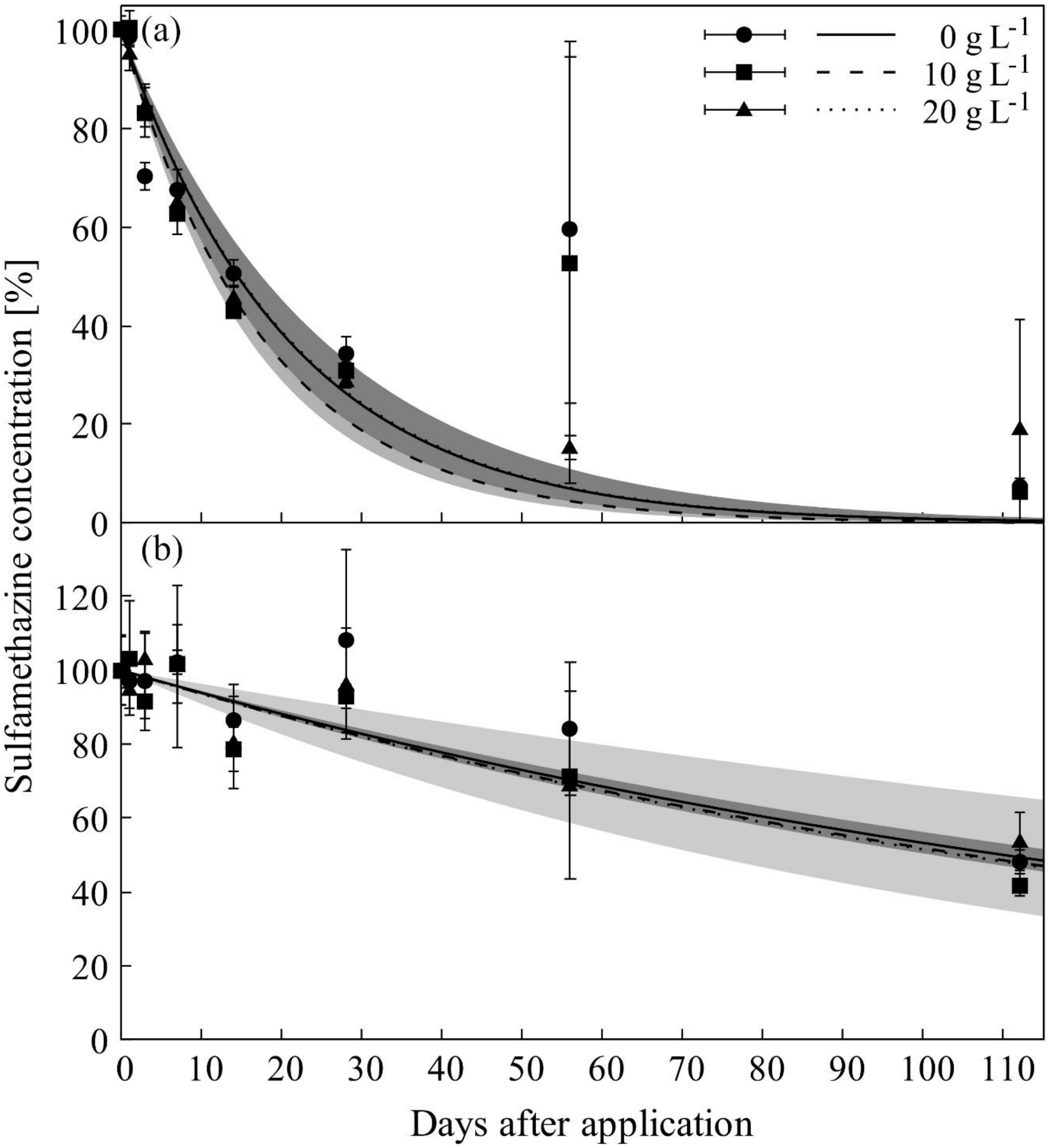

Supplementary Fig. S1 Dissipation of sulfamethazine in water (a) and soil (b) phase of flooded soil systems under different salt concentrations (0 g L^{-1}, 10 g L^{-1}, 20 g L^{-1}). Data points and error bars represent the mean of three replicates with standard deviation; lines represent fitted dissipation curves; calculated borders of asymptotic standard error were grey deposited for the particular salinity levels

Supplementary Tab. S3 Physico-chemical properties of the selected veterinary antibiotics

**Antibiotics**	**pK$_{a1}$**	**pK$_{a2}$**	**K$_d$**	**log *K$_{ow}$***	**Reference**
SDZ	2.00	6.40	1.4-2.8 [a]	-0.09 [a]	[1]
SMZ	2.60	8.00	0.66-6.73 [b]	0.28 [d]	[2]
SMX	1.60	5.70	1.5 [c]	0.89 [e]	[2]
TMP	3.23	6.76	9.7 [c]	0.91 [e]	[3]

SDZ, sulfadiazine; SMZ, sulfamethazine; SMX, sulfamethoxazole; TMP, trimethoprim
[a] [4] [b] [5] [c] [6] [d] [7]

[e] [8]

pK$_{a1-4}$: negative decimal logarithm of acidic dissociation constant

K$_d$: soil-water partitioning coefficient [L kg^{-1}]

log K_{ow}: decimal logarithm of the octanol-water partitioning coefficient

Supplementary Tab. S4 Apparent distribution coefficient (K$_{app}$) of sulfadiazine (SDZ), sulfamethazine (SMZ), sulfamethoxazole (SMX) and trimethoprim (TMP) (mean of three replicates) over the period of 112 days under different salt concentrations

**Antibiotic**	**Salt**	**Days of incubation**							
	[g L^{-1}]	0	1	3	7	14	28	56	112
	0	1.42	1.57	2.19	1.94	2.27	4.28	3.17	13.20
SDZ	10	1.82	1.84	1.85	2.66	2.70	5.07	4.68	23.57
	20	1.47	1.63	1.85	2.38	2.17	5.20	7.75	8.57
	0	3.08	3.03	4.27	4.68	5.31	9.66	5.80	20.80
SMZ	10	3.08	3.16	3.38	4.96	5.61	9.24	7.29	20.46
	20	2.67	2.66	3.26	4.12	4.68	8.95	12.22	15.96
	0	1.18	1.22	1.55	1.99	3.66	11.01	0.14	6.71
SMX	10	1.28	1.49	1.27	2.09	5.02	31.07	101.54	6.33
	20	1.21	1.30	1.54	2.18	13.52	14.28	28.84	27.01
	0	67.38	74.31	129.05	156.71	166.86	186.69	496.54	276.86
TMP	10	106.58	49.27	60.54	80.65	50.55	567.14	186.77	213.58
	20	30.11	32.63	48.57	52.77	70.49	77.32	143.83	293.00

References Supplementary Material

1. Leal, R. M. P., Alleoni, L. R. F., Tornisielo, V. L. & Regitano, J. B. Sorption of fluoroquinolones and sulfonamides in 13 Brazilian soils. *Chemosphere* **92,** 979–985 (2013).

2. Ikehata, K., Jodeiri Naghashkar, N. & Gamal El-Din, M. Degradation of Aqueous Pharmaceuticals by Ozonation and Advanced Oxidation Processes: A Review. *Ozone Sci. Eng.* **28,** 353–414 (2006).

3. Qiang, Z. & Adams, C. Potentiometric determination of acid dissociation constants (pKa) for human and veterinary antibiotics. *Water Res.* **38,** 2874–2890 (2004).

4. Thiele-Bruhn, S., Seibicke, T., Schulten, H.-R. & Leinweber, P. Sorption of sulfonamide pharmaceutical antibiotics on whole soils and particle-size fractions. *J. Environ. Qual.* **33,** 1331–1342 (2004).

5. Chu, B., Goyne, K. W., Anderson, S. H., Lin, C.-H. & Lerch, R. N. Sulfamethazine Sorption to Soil: Vegetative Management, pH, and Dissolved Organic Matter Effects. *J. Environ. Qual.* **42,** 794–805 (2013).

6. Liu, F. *et al.* Dissipation of sulfamethoxazole, trimethoprim and tylosin in a soil under aerobic and anoxic conditions. *Environ. Chem.* **7,** 370 (2010).

7. Martin, Y. C. Exploring QSAR. Hydrophobic, Electronic, and Steric Constants C. Hansch, A. Leo, and D. Hoekman. American Chemical Society, Washington, DC. 1995. Xix + 348 pp. 22 × 28.5 cm. Exploring QSAR: Fundamentals and Applications in Chemistry and Biology . C. Hansch and A. Leo. American Chemical Society, Washington, DC. 1995. Xvii + 557 pp. 18.5 × 26 cm. ISBN 0-8412-2993-7 (set). $99.95 (set). *J. Med. Chem.* **39,** 1189–1190 (1996).

8. Lin, K. & Gan, J. Sorption and degradation of wastewater-associated non-steroidal anti-inflammatory drugs and antibiotics in soils. *Chemosphere* **83,** 240–246 (2011).
